# Review of Recurrent Otomycosis and Clotrimazole in Its Treatment

**DOI:** 10.7759/cureus.30098

**Published:** 2022-10-09

**Authors:** Mehreen Haq, Prasad Deshmukh

**Affiliations:** 1 Otolaryngology, Jawaharlal Nehru Medical College, Datta Meghe Institute of Medical Sciences, Wardha, IND; 2 Otolaryngology - Head and Neck Surgery, Jawaharlal Nehru Medical College, Datta Meghe Institute of Medical Sciences, Wardha, IND

**Keywords:** infection, clotrimazole, treatment, otomycosis, recurrent

## Abstract

Otomycosis is a disease whose acute form affects four in 1,000 persons annually and the chronic form affects 3-5% of the population. It is brought on by various fungi, primarily saprophytes which most commonly include the Candida albicans and Aspergillus niger. The disease rarely poses a life-threatening danger, but as it requires prolonged treatment and follow-up and has a significant chance of recurrence, it has a difficult and taxing course. Numerous therapeutic modalities are available for the treatment of otomycosis. In the beginning, the fungal elements are removed by ear toilet - washing or suctioning of the ear canal followed by drying. Topical therapy includes the use of antifungals, of which the most commonly used drugs include clotrimazole or miconazole, often given along with ceftazidime which is an antibacterial. The primary mechanism by which clotrimazole works is by impairing the permeability barrier of the cytoplasmic membrane of the fungi, which causes holes to appear in the cell membrane and leaking out of the contents of the organism, thus killing the fungus and treating the infection. Various studies suggest that following clotrimazole drop therapy, symptoms suggestive of otomycosis are not at risk for recurrence of the disease and due to its economical pricing and easy availability, is frequently recommended by otolaryngologists in the treatment of otomycosis. In this review article, we will discuss about the effectiveness of the drug in different populations, observe treatment failures and relapse of the disease, analyze the ability of clotrimazole drops in preventing relapse of the infection, and observe the role of the drug in reducing the signs and symptoms of the disease.

## Introduction and background

One of the more widespread infections in the world is otomycosis, a disease that affects the external auditory canal (EAC). It primarily affects people who live in hot, dusty, and humid tropical and subtropical regions [1,2]. The occurrence of fungal infection is not only affected by geographical distribution, but also varies with distance and time [3]. Predisposition to the development of otomycosis includes tampering with the ears, increased temperature, dampness, age, bacterial infections that may lead to secondary infections, steroid use, injury, swimming, and immunological abnormalities [4,5]. Numerous microbes are responsible for causing the infection but the most common saprophytes isolated from cultures include Aspergillus niger and Candida albicans [6]. A flavus and A fumigatus are the other important organisms of the aspergillus species which play an important role in the progression of this disease [7]. The other fungal agents include Cladosporium spp., Alternaria spp., Mucor spp., and Rhizopus spp. Dermatophytes have also been reported to cause the disease in very rare incidents [8].

The infection is typically unilateral and is characterized by otalgia, scaling, loss of hearing, erythema and acute pruritis, affecting people of all age groups ranging from infants to adulthood (81 years) with the average age being 30-40 years. Males and females are equally affected and there is no significant difference in the occurrence of the infection in rural and urban areas [1]. Diagnosis of the infection is made by collecting samples of discharge from the affected ear, wax and scrapings of scales present in the canal and detecting various fungal details, namely mycelium, pseudo-mycelium and yeast in the various samples. Aspergillus niger appears as having a long white filamentous hypha with small black conidiospores. Blue, black, bluish-green, yellow and white fungal growth and debris is seen on examination of the external ear [9].

Otomycosis has historically been treated with a variety of topical antifungal medications, either alone or in combination. Eliminating observable debris and fungal components is the mainstay of therapy for the infection. Apart from this, steroids, acidic solutions, antiseptics, and antifungal medications are some of the topical therapeutic agents that are recommended for the control of this illness. Antifungal medications are frequently used to treat ear fungal infection, however, the condition is not always cured. The physiological signs of the external ear canal should also be improved by the infection's treatment [10,11]. Specific topical antifungal includes drugs belonging to azole groups and other group of drugs such as clotrimazole, nystatin, tolnaftate, miconazole, econazole, acetic acid, potassium sorbate and non-specific medications comprising Boric acid, m-cresyl acetate, alcohol and gentian violet. Clotrimazole has been found to be 83% effective according to a study [12]. Its usage has also been proven to reduce the recurrence of the disease and the low cost of the drug in the market in comparison to the high cost of treatment of the disease makes it a suitable drug for the treatment of otomycosis and for the prevention of relapse of the disease [1].

## Review

Pathophysiology and clinical features

According to reports, otomycosis can occur in as little as 9% of otitis externa cases and as many as 30.4% of cases, considering that 30.4% of individuals present with symptoms of inflammation or otitis health issues with the ears [13]. Otitis media patients may have fungal colonization of the external auditory canal if there is prolonged discharge and maceration of the epithelium. Conidiophores in the outer ear indicate that the fungi utilize the discharged mucus secretion as a food source. The middle ear inflammation brought on by chronic hyperplasia of the mucous membrane is comparable to upper respiratory tract inflammation. Goblet cell metaplasia, increased mucus production, obstruction of mucociliary clearance, persistent mucosal inflammation with lymphocytes and plasma-cell inflammation, and fibrosis are all characteristics of this infection. As a result, the tympanic membrane perforates, and the continuous outflow of fluids from the middle ear cavity to the auditory tube is disrupted [14-16]. Inoculation of fungus in most medial regions of the external canal, or direct disease extension from nearby skin is likely to result in tympanic membrane involvement [17].

Clinical features of otomycosis are nonspecific which present as pruritis, ear pain and discomfort, auditory fullness, tinnitus, hearing loss, and occasionally discharge. Recurrence is also a common symptom, which is exceedingly frustrating for both the patient and the doctor as it necessitates ongoing treatment and monitoring [13]. According to various studies, the most common clinical symptom comprises pruritis/itching (74%) and otalgia (60%) followed by a blocked sensation in the ear (50%); other symptoms involve hearing loss (44%), discharge (36%), tinnitus (8.9%), inflammation, otorrhea and scaling [18-22]. Table 1 shows the same.

**Table 1:** Common clinical symptoms of recurrent otomycosis

Pruritis	74%
Otalgia	60%
Blocked sensation	50%
Hearing loss	44%
Discharge	36%
Tinnitus	8.9%

Treatment with clotrimazole and other modalities

The initial step in treatment is to carefully dry and clean the external auditory canal, ideally using suction evacuation. Syringing the external auditory canal should be prevented since it can occasionally cause the infection to spread to deeper anatomical areas, especially if the tympanic membrane is perforated and obscured by waste material that has damaged it. If suction evacuation facilities are not available, regular saline along with antifungal powder should be syringed under all aseptic circumstances. After syringing, the ear should be thoroughly dried off since moisture encourages fungi to continue growing. Methylated spirit can be used to dry mop the ear, albeit it might be slightly uncomfortable [23]. Applying antifungal ointment to the external auditory canal is the alternative therapeutic method. Mercurochrome, an organic mercurial compound that is water soluble, is frequently used as an antibacterial agent and is also known to have antifungal effects when applied topically. With a reported efficacy range between 96% and 100%, it is frequently used as a 1-2% solution and has been employed specifically in situations with humid surroundings [24].

Clotrimazole: mechanism of action

Clotrimazole belongs to the imidazole class of drugs. It is a broad-spectrum antimycotic and is most commonly used for the treatment of candida and other fungal infections. It is a synthetic azole, which can be utilized in common topical treatment for vulvovaginal candidiasis, oropharyngeal candidiasis, skin infections by dermatophytes and various fungal infections. It exhibits fungistatic antimycotic action by concentrating on ergosterol production and biosynthesis, thereby preventing the growth of fungi [25]. Additionally, it exhibits some in vitro action against specific Gram-positive bacteria and exhibits activity against Trichomonas species at very large concentrations [22]. The main way that clotrimazole works is by impairing the permeability barrier in the cytoplasmic membrane of the fungus. By preventing the demethylation of 14 alpha lanosterol, clotrimazole consequently suppresses the manufacture of ergosterol in a concentration-dependent manner. The cell can no longer build an unbroken and an efficient cell membrane when ergosterol synthesis is suppressed [26-28]. Because ergosterol also directly stimulates the growth of fungal cells in a hormone-like manner, the commencement of the aforementioned events occurs quickly, which inhibits fungal growth in a dose-dependent manner. Clotrimazole has other pharmacological effects in addition to inhibiting ergosterol manufacture, which is how it works against fungi. The sarcoplasmic reticulum Ca2+ ATPase is inhibited, intracellular calcium levels are decreased, and calcium-dependent potassium channels and voltage-dependent calcium channels are blocked. Clotrimazole's effects on other cell targets outside of its antimycotic activity can be attributed to this action [29,30].

Clotrimazole is available in various formulations under innumerable FDA-approved names which include powders, topical lotions, oral lozenges and vaginal inserts/ tablets. It can be administered by oral as well as topical routes. Cream, ointment and lotion formulations are for topical administration; gently massage cream or solution on the cleansed, afflicted skin. Topical medications shouldn't be applied intravenously or to the eye. Other oral formulation includes transmucosal delivery. Troches should be dissolved in the mouth gradually; patients should not chew them.

Adverse effects and contraindications of clotrimazole

Itching, nausea and vomiting are some of the detrimental effects of the oral formulation. The oral formulation may cause abnormal liver function tests in more than 10% of patients. For this reason, while using oral clotrimazole (troche), there should be periodic monitoring of the liver profile. There is a typical complaint of burning by patients when using clotrimazole to treat fungal infections. Rashes, hives, blisters, burning, stinging, peeling, redness, swelling, discomfort, or other indications of skin irritation are some additional side effects. If irritation or sensitivity appears at the administration site, topical formulations should only be applied externally, and should be stopped immediately [31].

The drug is contraindicated in various conditions which may include:

Pregnancy

Following cutaneous or intravaginal dosing, clotrimazole exhibits limited absorption. During pregnancy, only topical medications are advised. However, there is no evidence that clotrimazole crosses the placenta. The FDA has categorized clotrimazole as a class C medication for pregnancy risk. Pregnant women who use oral clotrimazole have not been the subject of an adequate, controlled investigation. After the usage of clotrimazole medication in pregnancy, there have been no teratogenic consequences shown [32,33].

Breast Feeding

There is no research on clotrimazole being used while nursing. Regarding clotrimazole's excretion in breast milk, there is no information available. Since topical clotrimazole is not anticipated to significantly affect maternal absorption, it provides little threat to nursing infants [34].

Azole Antifungals Hypersensitivity

Clotrimazole should not be used by patients who are hypersensitive to azole antifungals. The formulation elements found in certain clotrimazole formulations frequently cause hypersensitivity responses [25].

Drug-Drug Interactions

Excessive use of clotrimazole may lead to a significant increase in the level of tacrolimus, leading to toxicities related to tacrolimus. Therefore, it is necessary to keep in check the amount of drug administered in the body [35]. When clotrimazole is used locally or topically, harmful side effects can include pelvic pains, hives, skin rash, sporadic headaches, itching and irritation.

Effect of clotrimazole in recurrent otomycosis

Numerous studies have examined the efficacy of different antifungal medications for ear infections in both in vivo and in vitro settings [36]. In a clinical study conducted, 87 patients having otomycosis, showing non-bacterial elements were given a combination therapy of ceftizoxime and clotrimazole. After receiving clotrimazole cream and ceftizoxime powder, the clinical symptoms of the intervention group significantly improved, pain reduced from 77.8% in the first visit to 11.1% in the second, the swelling and itching also went down from 57.8% to 2.2% and 84.4% to 15.6% in the first and second visit, respectively. The discomfort and edema, however, significantly decreased in the control group from 69% to 52.5% [37]. In maximum clinical studies, for the management of otomycosis, the most frequently used topical azole is clotrimazole. Most studies suggest a reported rate of efficiency that ranges from 95% to 100%, hence it looks to be among the most efficient treatments available for otomycosis [38-41], except for one study that reported a lower effectiveness rate of 50% [41]. When treating complex bacterial and fungal infections, clotrimazole's antibacterial activity is a bonus. It is thought to have no ototoxic effects. Clinical indications of clotrimazole ototoxicity have not been reported [42]. In another descriptive study conducted in Hyderabad, 1% solution of clotrimazole was attributed to be extremely effective to accomplish clinical cure in 191 (92.27%) patients within a short time span of 1-2 weeks [43]. Yet another study revealed that in 96 percent of instances, topical clotrimazole cream use combined with mechanical debridement led to an immediate remission of symptoms [20]. In a case report by Jackman et al., clotrimazole also exhibited the highest efficacy among initial antifungal drug therapy [41]. Stern et al. discovered that clotrimazole was the most efficient treatment for typical fungal species in in vitro experiments. Clotrimazole is the most effective in vivo antifungal drug, followed by gentian violet and nystatin, according to additional, clinical investigations [4,36]. The results of another clinical trial following the end of the treatment cycle revealed that on the fourth day after therapy, 55.6% and 58.8% of patients treated with betadine and clotrimazole, respectively, exhibited partial responses to treatment. Additionally, on the tenth day following treatment, 46.1% of patients receiving clotrimazole had an excellent reaction and 49.1% of patients receiving povidone-iodine had a partial response. Finally, 68.6% of patients who were treated with betadine and 66.7% of patients who were treated with clotrimazole showed a favourable response to treatment on the twentieth day following treatment [11]. Another study identified clotrimazole and econazole as the preferred medications for treating otomycosis [41]. On 40 otomycosis patients, Dundar and İynen did a prospective research. In this trial, an intravenous catheter and syringe were used to inject 1% clotrimazole into the ear canal. The authors described how well a single dose of clotrimazole, 1%, worked to treat otomycosis [44]. Major relapse cases were seen in patients with irritated or ulcerated tympanum and canal. Clotrimazole drop may be used to stop the relapse of otomycosis in persons who have this condition, particularly those with ear and tympanic abnormalities [1].

## Conclusions

Otomycosis is rarely fatal, but it can be difficult to treat and follow up because of its long-term treatment and high recurrence rate. This can be frustrating for both patients and otolaryngologists. Although this disease is caused by a large number of organisms, Aspergillus niger is the most common fungi affecting the external auditory canal of the patient leading to severe pruritis and otorrhea. The infection is characterized by maceration of the ear canal with severe discharge, inflammation and redness of the external auditory canal, goblet cell metaplasia, and fibrosis, in severe cases, tympanic membrane perforation and relapsing otorrhea are seen. Historically this infection has been treated by a variety of treatment modalities which include antifungal drugs, steroids and oil solutions but the most important prophylaxis includes keeping the ear clean from all the debris and maintaining dryness of the ear for the early cure of the disease. Among the antifungal drugs, clotrimazole belonging to the imidazole group of classification is considered to be one of the most efficient drugs due to its 95-100% efficacy in treating the disease. Results of various studies prove that clotrimazole is an excellent drug for treating the infection as it helps in a significant reduction in the signs and symptoms of the disease in a short span of time and also reduces the chances of relapse, hence it can also be used in the prevention of recurrence of the disease. It is the preferred drug in all cases of otomycosis due to its mechanism of impairing the cell membrane and decreasing the production of ergosterol thereby inhibiting the growth of the fungi in a dose-dependent manner. Thus, the drug has a significant role in the treatment of recurrent otomycosis.
